# Efficacy of bone morphogenetic protein in comparison with autogenous bone in regeneration of ameloblastoma bone defects. A systematic review

**DOI:** 10.4317/medoral.26729

**Published:** 2024-10-13

**Authors:** Willmar Yeraldo Pelegrin-De-Los Santos, Eloy Benito-Ramal, Jorge Toledano-Serrabona, Mª Ángeles Sánchez-Garcés

**Affiliations:** 1 Dental Student. School of Medicine and Health Sciences, Campus de Bellvitge, Universitat de Barcelona, Spain; 2 DDS, MS, PhD. Clinical Assistant Professor of Oral Surgery and Implantology, School of Medicine and Health Sciences, Campus de Bellvitge, Universitat de Barcelona. Researcher of the IDIBELL Institute, Barcelona, Spain; 3 MD, DDS, MS, PhD, EBOS, Lecturer in Oral Surgery and Implantology. School of Medicine and Health Sciences, Campus de Bellvitge, Universitat de Barcelona. Researcher of the IDIBELL Institute, Barcelona, Spain

## Abstract

**Background:**

To evaluate the evidence comparing bone morphogenetic proteins (BMPs) and autogenous bone grafts (ABGs) for regenerating bone defects from ameloblastoma.

**Material and Methods:**

An electronic search was performed in PubMed and Scopus from October to December 2023, supplemented by manual searches and review of relevant study reference lists. Cohen’s kappa was calculated to assess the interrater reliability between two independent investigators. The methodological quality and risk of bias of the selected articles was assessed using the JBI checklist for case series and the NOS for observational studies.

**Results:**

Nine studies met the inclusion criteria and were selected for the qualitative synthesis. Cohen’s kappa (κ) value resulted in 98.21% agreement. A total of 229 participants were included. The BMPs were evaluated in five studies, and four evaluated the ABGs. The BMPs has been tested in 25.76% of the patients, while ABGs were studied in 74.24%. In order to evaluate the final result of regeneration, all the studies based their analysis on postoperative questionnaires, radiographical (CBCT and/or panoramic) and/or clinical examination. The results showed a higher regeneration success rate in the studies where the BMPs was used.

**Conclusions:**

Considering the limitations of the studies and the review, it has been shown that BMPs may yield favorable outcomes in terms of bone regeneration, as compared with ABGs.

** Key words:**Ameloblastoma, reconstruction, bone defect, graft, autogenous bone, bone morphogenetic protein, BMP.

## Introduction

Ameloblastoma is a rare benign epithelial odontogenic tumor that constitutes about 10% of all tumors that arise in the jawbones (1). It typically affects individuals aged 30-60 and is more prevalent in Africa, China and India compared to the Western world (2). According to the 2022 WHO classification (3), ameloblastoma presents in five types, all originating from odontogenic epithelium, such as cell rests of the dental lamina, enamel organ, the lining of an odontogenic cyst or the basal cells of the oral mucosa, and mostly occur in the mandible (4). These tumors are generally characterized by painless, progressive growth leading to facial asymmetry, tooth displacement, and possible fractures.

Diagnosing typically ameloblastoma requires CT imaging and biopsy due to the tumor’s intricate presentation (1). Treatment options range from less invasive procedures such as enucleation and marsupialization, to more aggressive approaches such as resection with a safety margin and immediate bone reconstruction to help speech and swallowing. The latter is the standard treatment. NoTable, radical resection significantly reduces recurrence rates compared to more conservative treatments (5).

The challenge of reconstruction significant resection defects is appreciable, particularly due to the variability in bone loss (6). Reconstructive options include autogenous bone grafts (ABGs) (7), the usage of bone morphogenetic proteins (BMPs) (8), among other suiTable options such as allografts, combination of allografts and autografts, alveolar distraction osteogenesis, segmental bone transport, customized implants, etc. (9,10). BMPs, especially recombinant types like rhBMP-2, play crucial roles in bone and cartilage formation and are used with carriers to enhance controlled release and minimize complications like ectopic calcification (8).

Despite ABGs being the traditional gold standard for reconstructing facial bone defects, it carries risks and limitations such as donor site pain, increased surgical time and insufficient bone graft material (11). The development of effective reconstruction techniques using osteoinductive elements, such as growth factors (GFs), without the need for traditional bone grafting, has the potential to mitigate surgical morbidity. The objective of the present systematic review was to analyze the latest scientific evidence to compare the efficacy of BMPs with traditional autologous bone grafts in regenerating bone defects caused by ameloblastoma.

- Abbreviations

ABGs: Autogenous bone grafts; ABP: Autogenous bone particles; AIC: Anterior iliac crest bone grafts; BMPs: Bone morphogenetic proteins; CBCT: Cone beam computed tomography; CS: Case Series; DBM: Demineralized bone matrix; F: feminine; FALM: Frozen autogenous lesioned mandible; FDA: Freeze dried allograft; GFs: growth factors; IC: Iliac crest; M: masculine; NVBGs: Non-vascularized bone grafts; OHRQL: Oral health related quality of life; PDLLA: poly(D,L-lactide); PIC: Posterior iliac crest bone grafts; PLLA: poly(L-lactide); PRP: Platelet-rich plasma; QOL: Quality of life; rhBMP-2: recombinant bone morphogenetic protein 2; RS: Retrospective Study; SD: Standard deviation; T: Total; TBG: Tibia bone graft; VABT: Vascularized autogenous bone transplant.

## Material and Methods

- Study design and research question

This systematic review was carried out based on the Preferred Reporting Items for Systematic Reviews and Meta-Analyses (PRISMA) guidelines (12). The following PICOS question (Population, Intervention, Comparison, Outcome and Study type) was prepared:

Population: Patients of all ages with a diagnosis of an ameloblastoma and treated with a maxillary or mandibular resection.

Intervention: Bone reconstruction with BMPs carrier.

Comparison: Bone reconstruction with ABGs.

Primary outcome: Radiographical and clinical outcomes: Bone continuity, preservation and stability using an objective method (such as CBCT imaging and/or panoramic radiographs) and adequate healing / complete closure of intraoral and/or extraoral wounds.

Secondary outcome: surgical time, complications during surgery and/or in the follow-up period, quality of life (function and aesthetic), recurrence rate and cost.

Study type: Original, human randomized and non-randomized clinical trials, observational studies (prospective and retrospective) and case series.

- Eligibility criteria

The inclusion criteria were clinical trials, observational studies and case series with a minimum 10 patients, being made on humans which analyse the outcomes within an interval between 6 months and 10 years of follow-up and published in English or Spanish within the last 23 years (2000-2023). The exclusion criteria were systematic reviews and meta-analyses, case reports, observational and clinical studies and case series with less than 5 ameloblastoma patients, book chapters, ex-vivo studies, studies in animals and studies that analyse other type of reconstruction technique, other purposes, other type of tumour and other ameloblastoma surgical techniques.

- Search strategy

An electronic search was conducted in PubMed (MEDLINE) and Scopus databases between October and December 2023. The following keywords have been used in the respective search engines: “ameloblastoma”, “reconstruction”, “bone defect”, “graft”, “autogenous bone”, “bone morphogenetic protein” and “BMP”, combined with the Boolean operators “AND” and “OR”: ((((ameloblastoma) AND (reconstruction)) AND (bone defect))) AND (graft)))) AND (((autogenous bone) OR (bone morphogenetic protein)) OR (BMP))). In addition, a manual search was performed in Google Scholar and the most relevant journals: Journal of Craniofacial Surgery, Baylor University Medical Center Proceedings, Annals of Maxillofacial Surgery, Craniomaxillofacial Trauma Reconstruction, JAMA Facial Plastic Surgery, Oral Surgery Oral Medicine Oral Pathology and Oral Radiology and Journal of Oral and Maxillofacial Surgery. References of the relevant studies were also examined to identify other articles. The last search was performed on December 17, 2023.

- Selection of studies

First, all those duplicate articles have been removed. Successively, those showing a title and an abstract of interest have been selected, and the resulting registers have been evaluated for full text eligibility. Two independent investigators (W.P.D & E.B.R), according to the pre-established inclusion and exclusion criteria, independently screened the full text of every article in order to establish its eligibility. A Cohen kappa was calculated (J.T.S) to determine the interrater reliability. The three reviewers resolved any disagreements.

- Data extraction and method of analysis

The two reviewers carry out the data extraction of the selected articles. The characteristics collected from the studies to perform a qualitative analysis were author data, year of publication, country, type of study, nº of ameloblastoma and total patients, age (mean ± standard deviation or age range), sex, and characteristics based on the ameloblastoma tumor such as location, surgical treatment and size of the defect, type of ABGs, type and dose of BMPs used, type of BMPs carrier and graft extender used, method of measuring regeneration (clinical and/or radiographical), length of follow-up, complications and morbidity, quality of life, recurrence rate, surgery time, cost and success rates. Authors were contacted for clarification of missing or unclear information when it was necessary. The main outcomes of the analysis were the efficacy of bone volume preservation measured by CBCT imaging and panoramic radiographs, complications, surgery time and cost. The methods used to present and synthesise the results were Tables, Figures and descriptive measures.

- Quality and risk of bias assessment

The methodological quality of the selected articles was assessed by the two reviewers using the Joanna Briggs Institute critical appraisal tools (JBI) (13) for cases and case series and the Newcastle-Ottawa scale (NOS) for observational studies. In case series, a response of ‘no’ to any of the questions negatively impacts the quality of the case series. These scales were answered by two independent investigators (W.P.D & E.B.R), and disagreements were resolved by discussion between them.

## Results

- Search and study inclusion

**Figure 1:**
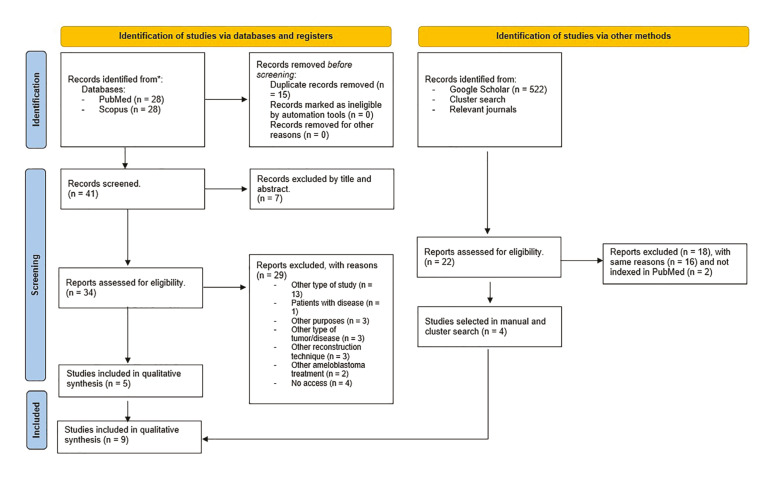
Study selection process summarized in the PRISMA flowchart.

According to the PRISMA guidelines, Fig. 1 illustrates the flow diagram of the study selection process (12). The initial electronic search yielded 56 articles with 41 remaining after duplicate removal. Titles and abstracts were reviewed, leading to the selection of 34 articles for full text evaluation. After applying exclusion criteria, 29 publications were discarded. Finally, and after a manual search and examination of the relevant studies, 9 studies (7,14-21) met the inclusion criteria and were selected for the qualitative synthesis. The reliability of the study selection process was estimated by calculating an inter-agreement score and Cohen’s kappa (κ) value, resulting in 98.21% agreement and κ = 0.9453, indicating almost perfect agreement.

- Assessment of methodological quality: risk of bias of included studies

A critical analysis using NOS and JBI checklist revealed a high quality and low risk of bias in observational studies (15-17,19,21). For case series, Simon *et al*. (14) showed a high risk, while several studies (7,18,20) exhibited moderate (7,20) and low risk of bias (18), respectively. See in Table 1 and Table 2.

- Characteristics of included studies

The included studies, published between 2006 and 2024, originated from five different countries and involved a total of 229 ameloblastoma participants, with case sizes ranging 5 to 136 patients. Due to incomplete data, mean age and gender percentages were not calculated. The studies primarily reported ameloblastomas in body, angle, ramus, symphysis and/or parasymphysis (7,14,15,18-21). All studies described ameloblastoma resections and various reconstruction techniques, with defect sizes ranging from 1 to 14 cm. Follow-up varied from a minimum 1 month to a maximum 80 months, with only cases having at least 6 months of monitoring selected.

The main results of the included studies are described in Table 3 and Table 4.

- Efficacy of regeneration with BMPs grafts vs autogenous grafts in ameloblastoma bone defects

BMPs were evaluated in 5 studies (17-21) covering 25.76% of the patients, while ABGs were studied in 74.24% across 4 studies (7,14-16). The most frequently studied BMP was rhBMP-2, employed in various carriers such as ABGs, particulate marrow, allogenic bone, bioceramics, among others. Melville *et al*. (18) was the only study to report specific dosages, ranging from 8.4 to 12.0 mg. Regeneration outcomes were assessed via postoperative questionnaires, radiographical (CBCT and/or panoramic) and/or clinical examination, with implant prosthetic rehabilitation considered a critical success factor in 6 studies (7,16-18,20,21).

Studies using ABGs showed a greater variety of complications and morbidity compared to those using BMPs, with no tumor recurrences reported in the latter group. Only three studies (17,18,21) provided data on cost and surgery time. The success rate for bone regeneration ranged from 63.6% to 90% with ABGs, and from 80% to 100% with BMPs, despite, the high heterogeneity of different grafts and carriers used (Table 4).

## Discussion

The present systematic review critically evaluates the comparative efficacy of BMPs and ABGs in regenerating bone defects following ameloblastoma resection. Our findings suggest that BMPs, particularly rhBMP-2, may offer superior outcomes in terms of bone regeneration success rates and fewer postoperative complications comparted to ABGs.

The potential benefits of GFs such as the BMPs in bone regeneration can significantly enhance patient quality of life by achieving similar or better regeneration success rates than ABGs, with fewer complications and comparable opportunities for dental implant prosthesis rehabilitation. Although ABGs have been the gold standard for reconstructing mandibular defects, the ability to achieve successful outcomes while avoiding the comorbidity associated with autogenous bone donor sites is an important consideration.

BMPs, with success rates ranging 80-100%, have proven to be effective osteoinductive agents. These proteins promote osteoblast differentiation and enhance bone repair, aligning with the literature that supports their use in managing complex bone defects. Notably, the use of rhBMP-2 has shown particular promise, although it also poses risks such as the potential ectopic bone formation if not properly controlled.

In contrast, the studies involving ABGs have demonstrated a wider range of success rates (63.6% to 90%) and a higher incidence of complications. This variability is attributed to factors such as donor site morbidity, limited tissue availability, and the inherent complexities of grafting procedures. These findings corroborate previous research indicating that while ABGs are effective, they carry significant drawbacks (7,14-16).

The lower incidence of complications with BMPs use is noTable, suggesting that BMPs may offer a safer alternative to ABGs, specially in complex clinical scenarios where minimizing patient morbidity is crucial. Furthermore, the economic aspects of BMPs versus ABGs use, including cost-effectiveness and surgery duration, were not adequately explored due to insufficient data. This represents a significant gap in the literature, as cost considerations are crucial in the broader implementation of new medical technologies.

Only one other systematic review has been identified that evaluates the use of GFs for mandibular defects, which reported a slightly lower success rate for BMPs (86.5%) compared to our findings (11). This discrepancy could be due to different study inclusion criteria or variations in BMPs formulations and delivery methods used across studies.

This systematic review encountered several limitations. First, one of the selected articles showed a high risk of bias or poor quality (14). Second, the comparison across studies was limited by a variety of sample sizes, types of regenerations, carriers, grafts, the absence of control groups, and the population scrutinized. These confounding variables prevent us from drawing statistically reliable and robust conclusions about which BMP carrier is more efficacious in bone regeneration. Furthermore, the success rate reported by some studies do not specifically pertain to patients with ameloblastoma, adding to the uncertainty of the evidence (7,15,16,18).

In conclusion, while BMPs showed promise in the regeneration of ameloblastoma-related bone defects, further research is needed to optimize their delivery and to fully understand their long-term outcomes compared to ABGs. More clinical trials with rigorous methodologies and longer follow-up periods are required to confirm these preliminary findings and to establish more definitive guidelines for their use in clinical practice.

## Figures and Tables

**Table 1:** Assessment of the risk of bias and the methodological quality of the included case series studies according to JBI critical appraisal tool for Case Series.

Case Series	Q1	Q2	Q3	Q4	Q5	Q6	Q7	Q8	Q9	Q10	Overall risk determined by authors
Simon *et al.* (2006), Tanzania (14)	YES	NO	NO	NO	NO	YES	NO	YES	YES	YES	HIGH
Donkor *et al.* (2006), Ghana (7)	YES	YES	YES	YES	UNCLEAR	YES	NO	YES	YES	UNCLEAR	MODERATE
Melville *et al.* (2020), USA (18)	YES	YES	YES	YES	YES	YES	YES	YES	YES	YES	LOW
Clokie & Sándor (2008), Canada (20)	YES	YES	YES	NO	YES	YES	UNCLEAR	YES	UNCLEAR	UNCLEAR	MODERATE

Q1: Were there clear criteria for inclusion in the case series? Q2: Was the condition measured in a standard, reliable way for all participants included in the case series? Q3: Were valid methods used for identification of the condition for all participants included in the case series? Q4: Did the case series have consecutive inclusion of participants? Q5: Did the case series have complete inclusion of participants? Q6: Was there clear reporting of the demographics of the participants in the study? Q7: Was there clear reporting of clinical information of the participants? Q8: Were the outcomes or follow up results of cases clearly reported? Q9: Was there clear reporting of the presenting site(s)/clinic(s) demographic information? Q10: Was statistical analysis appropriate?

**Table 2:** Assessment of the risk of bias and the methodological quality of the observational studies according to NOS.

	Selection	Comparability	Outcome	Conclusion
Li et al. (2007), China (15)	***	**	***	Low risk of bias
Schlieve et al. (2015), USA (16)	***	**	***	Low risk of bias
Marechek et al. (2019), USA (21)	***	**	***	Low risk of bias
Marschall et al. (2020), USA (17)	***	*	***	Low risk of bias
Dastgir et al. (2024), USA (19)	***	**	***	Low risk of bias

**Table 3:** Description of the studies included in the systematic review.

Author (year), country	Type of study	No. of ameloblastoma patients (Total patients) [Mean age in years ± SD]/ Age range	Gender	Type of ameloblastoma and location	Surgical treatment received	Size of the defect in cm [Mean]	Follow-up in months [Mean]
Simon *et al.* (2006), Tanzania (14)	CS	11 [27]/17-47	6 (M) & 5 (F)	Multilocular lesion Body, angle, symphysis and/or ramus	Ablative surgery (segmental resection) followed by reconstruction of the mandible.	5.5-9.0	12-24
Donkor *et al.* (2006), Ghana (7)	CS	10 (T:29) [35.5]/12-65	18 (M) & 11 (F)	Angle, body	Trauma or elective surgery and reconstruction with rib grafts.	--	12
Li *et al.* (2007), China (15)	RS	136 (T:242) 9-73	156 (M) & 86 (F)	Lateral segment without condyle, anterior segment and combination	Resection of the lesioned mandible and reconstruction with different techniques.	6-14	1,3,6 &12
Clokie & Sándor (2008), Canada (20)	CS	9 (T:10) [43.1 ± 17.65]/18-73	3 (M) & 6 (F)	Body, ramus and anterior segment of the mandible	Resection of the lesioned mandible and reconstruction with BMP-7 and DBM and reconstruction plate.	3-9 [5.5]	9
Schlieve *et al.* (2015), USA (16)	RS	13 (T:20) [28.3]/9-63	11 (M) & 9 (F)	--	Transoral tumor resection & reconstruction with NVBG.	3-10.3 [6.43]	6-61 [22]
Marechek *et al.* (2019), USA (21)	RS	5 (T: 29) [55]/17-81	17 (M) & 12 (F)	Multilocular lesion Body, angle, symphysis and/or ramus	Secondary mandibular reconstruction with autogenous NVBGs (AIC or PIC). Autologous corticocancellous chips and cancellous marrow were then crushed and mixed with a BMP-impregnated collagen carrier. Fixation with reconstruction plate.	5-10 [7.2]	8-53 [26.6]
Marschall *et al.* (2020), USA (17)	RS	16 (T:47) [43 ± 16] / 13-70	24 (M) & 23 (F)	--	Tumor resection & Titanium reconstruction plate grafting with only a TBG or an AIC and using TBG + PRP + BMP + FDA.	[6.4 ± 1.6]	28
Melville *et al.* (2020), USA (18)	CS	19 (T:34) [37.79 ± 20.4]/9-89	19 (M) & 15 (F)	Body, ramus and anterior mandible.	Benign tumor ablation or trauma & reconstruction of the defect.	1.0-12.5 [5.5]	6
Dastgir *et al.* (2024), USA (19)	RS	10 (T:50) [51.71 ± 19.87] /17-83	--	Body, angle, condyle, symphysis and/or midline	Resection & reconstruction of mandibular segmental defects.	5-13 (T:3-20)	4-80 [21.70]

Abbreviations: SD: Standard deviation. RS: Retrospective Study. CS: Case Series. T: Total. F: feminine, M: masculine. PRP: Platelet-rich plasma. TBG: Tibia bone graft. AIC: Anterior Iliac bone graft. PIC: Posterior Iliac Crest bone graft. FDA: Freeze dried allograft. DBM: Demineralized bone matrix. NVBGs: Nonvascularized bone grafts.

**Table 4:** Analysis of the outcomes measured in the selected articles.

Author (year)	Type of autogenous bone graft / BMP carrier used	Type and dose of BMP used	Method of measuring	Complications percentage & most common complications	OHRQL & QOL	Morbidity	Recurrence rate	Cost	Surgery time in hours	Regeneration Outcome
Autogenous	
Simon *et al.* (2006) (14)		Reconstruction with: - Autogenous irradiated cortical scaffolds and ABP from anterior IC and PRP in 6 patients. - ABP taken from the anterior or posterior IC and PRP (without cortical scaffolds but with two plates in 5 patients.	Postoperative questionnaires and radiographs at 4,6 and 12 months.	36.4% Fractures (50%), plate traumatizing oral tissues (75%), recurrence (25%), loss of particulate bone chips (50%) and breakdown of the oral mucosa and infection (25%).	QOL: improvement in appearance & diminished pain.	--	1 (9%)	--	--	Success: 7/11 (63.6%) Failure: 4/11 (36.4%)
Donkor *et al.* (2006) (7)		Free non-vascularized autogenous rib grafts (with or without cartilage).	Considered to be successful if healing was uncomplicated within the first 6 months of its placement, and postoperative radiographic evaluation.	17.2% Wound dehiscence (6.9%) with exposure, graft resorption (3.4%) and recurrences (6.9%).	Improved appearance and easier prosthetic rehabilitation.	Complained of mild donor site pain.	2 (6.9%)	--	--	Success: 26/29 (89.7%) Failure: 3/29 (10.3%)
Li *et al.* (2007) (15)		Free autogenous bone transplant (group A), FALM (group B), FALM-iliac/rib compound (group C), VABT (group D), homologous bone transplant (group E), and hydroxylapatite (HA)/ titanium plate.	Clinical functional and aesthetic (facial symmetry, degree of mouth opening, occlusal relationship, and temporomandibular joint symptoms) and radiographic examination.	4.1% Local infection (50%), exposure of rigid fixation plate (20%), dehiscence (20%), and serious pain (10%).	Good functional and esthetic results were obtained in most patients.	Slight disfigurement of facial contour: 18/242 (7.4%)	--	--	--	Success: 203/242 (83.9%) Failure: 39/242 (16.1%)
Schlieve *et al.* (2015) (16)		Unicortical non-vascularized block of anterior iliac crest bone graft and titanium plate.	Considered to be successful if the bone reestablishment of mandibular continuity was sufficient for implant placement.	10% Local graft infection.	Most patients had maintenance of adequate bone for implant placement and functional dental rehabilitation.	Owing to the nature of this report, no comment on decreased morbidity because of this treatment method can be made.	Lesion recurrence rate was not included in the datasets.	--	--	Success: 18/20 (90%) Failure: 2/20 (10%)
BMP
Clokie & Sándor (2008) (20)	BMP-7 with 10 mL of DBM in a reverse-phase medium.	BMP-7	Clinical (manual manipulation) and panoramic radiographs evidence of restoration of mandibular continuity.	0%	Some patients had dental implants rehabilitation. Functional and aesthetic objectives were achieved.	--	--	--	--	Success: 9/9 (T:10) (100%)
Marechek *et al.* (2019), USA (21)	NVBGs (AIC or PIC). Autologous corticocancellous chips and cancellous marrow mixed with BMP-impregnated collagen carrier	rhBMP-2	Success was defined by radiographic evidence of bony continuity and stability at a minimum of 4 months postoperatively, as well as complete closure of intraoral and extraoral wounds.	40% Infection and graft failure.	Some patients had dental implants rehabilitation. Functional and aesthetic objectives were achieved.	Infection at the donor or recipient site, seroma, necessity for hardware removal, wound dehiscence, and fracture at the donor site.	--	--	Decreased operating time.	Success: 4/5 (80%) Failure: 1/5 (20%)
Marschall *et al.* (2020) (17)	Titanium reconstruction plate grafting with only a TBG or an AIC and using TBG + PRP + BMP + FDA.	rhBMP-2.	Graft success was defined as bony union demonstrated on panoramic radiographs and mandibular stability demonstrated on clinical examination at 4 months postoperatively.	--	Facial and mandibular bony reconstruction and dental prothesis rehabilitation.	Swelling.	--	The use of rhBMP-2 high costs ($5000 to $6000 for a large package).	--	Success: 15/16 (93.75%) Failure: 1/16 (6.35%)
Melville *et al.* (2020) (18)	Tissue-engineered composite grafts of allogeneic bone combined with rhBMP-2 and bone marrow aspirate concentrate. Nonresorbable titanium mesh or resorbable PLLA or PDLLA membrane.	8.4 - 12.0 mg of rhBMP-2	Clinical examinations and computed tomography scans were performed at 6 months postoperatively: 1.Bone union (homogenous radiopaque pattern continuous with native bone) without mandibular mobility. 2.Volume of grafted bone adequate for implant placement (at least 1.0 cm (height) by 0.8 cm (width)).	10% Mucosal dehiscence, cutaneous fistula and fracture.	Some patients had dental implants rehabilitation.	Decreased comorbidity.	--	Cost-efficient.	Decreases the length of the operation. Less time consuming.	Success: 27/30 (90%) Failure: 3/30 (10%)
Dastgir *et al.* (2024) (19)	Autogenous non-vascularized bone grafts: anterior iliac crest, costochondral graft, allografts in combination of BMP-impregnated collagen carrier.	--	Continuity of bone clinically and radiographically (CBCT) at a 4-month follow-up.	20% (T:34%) Infection and wound dehiscence.	--	Decreased comorbidity.	--	--	--	Success: 9/10 (90%) Failure: 1/10 (10%)

Abbreviations: BMP: Bone morphogenetic protein. rhBMP-2: Recombinant bone morphogenetic protein-2. OHRQL: Oral health related quality of life. QOL: Quality of life. PLLA: poly(L-lactide). PDLLA: poly(D,L-lactide). ABP: Autogenous bone particles. IC: Iliac crest. AIC: Anterior iliac crest bone grafts. PIC: Posterior iliac crest bone grafts. FALM: Frozen autogenous lesioned mandible. VABT: Vascularized autogenous bone transplant. DBM: Demineralized bone matrix. CBCT: Cone beam computed tomography. NVBGs: Nonvascularized bone grafts. TBG: Tibia bone graft. FDA: Freeze dried allograft. PRP: Platelet-rich plasma.
